# Effect of *Ficus pumila* L. on Improving Insulin Secretory Capacity and Resistance in Elderly Patients Aged 80 Years Old or Older Who Develop Diabetes After COVID-19 Infection

**DOI:** 10.3390/nu17020290

**Published:** 2025-01-15

**Authors:** Kenji Gonda, Takeshi Hai, Kouichi Suzuki, Akihiko Ozaki, Takashi Shibusa, Seiichi Takenoshita, Yuko Maejima, Kenjyu Shimomura

**Affiliations:** 1 Department of Breast and Thyroid Surgery, Jyoban Hospital of Tokiwa Foundation, Iwaki City 972-8322, Fukushima, Japan; ozakiakihiko@gmail.com; 2 Department of Gastrointestinal Tract Surgery, Fukushima Medical University, 1 Hikarigaoka, Fukushima City 960-1295, Fukushima, Japan; 3 Department of Bioregulation and Pharmacological Medicine, Fukushima Medical University, 1 Hikarigaoka, Fukushima City 960-1295, Fukushima, Japan; suzukou8931@gmail.com (K.S.); maejimay@fmu.ac.jp (Y.M.); shimomur@fmu.ac.jp (K.S.); 4 Department of Internal Medicine, Daido Central Hospital, 1-1-37 Asato, Naha City 902-0067, Okinawa, Japan; 81takeshi@gmail.com; 5 Department of Internal Medicine, Jyoban Hospital of Tokiwa Foundation, Iwaki City 972-8322, Fukushima, Japan; takashi-shibusa@tokiwa.or.jp; 6 Department of Drug Research for Astatine-221 Targeted Alfa Therapy, Fukushima Medical University, 1 Hikarigaoka, Fukushima City 960-1295, Fukushima, Japan; akiko-t@fmu.ac.jp

**Keywords:** *Ficus pumila* L., diabetes mellitus, COVID-19, HOMA-β, HOMA-IR

## Abstract

(1) Background: It has been reported that people affected by COVID-19, an infectious disease caused by SARS-CoV-2, suffer from various diseases, after infection. One of the most serious problems is the increased risk of developing diabetes after COVID-19 infection. However, a treatment for post-COVID-19 infection diabetes has not yet been established. In this study, we investigated the effects of *Ficus pumila* L. extract, which has traditionally been used to reduce blood glucose levels in Okinawa, on patients who developed diabetes after COVID-19 infection. (2) Methods: In total, 128 rehabilitation patients aged 80 years old or older who developed diabetes after COVID-19 infection were included. The HOMA-β (Homeostatic model assessment of β-cell function) and HOMA-IR (Homeostatic model assessment of insulin resistance) were assessed to evaluate the glucose tolerance. (3) Results: The HOMA-β decreased and HOMA-IR increased in patients who developed after diabetes after COVID-19 infection. Subsequently, 59 patients were given *Ficus pumila* L. extract and their HOMA-β and HOMA-IR improved after ingestion. On the other hand, the control group of patients who did not consume *Ficus pumila* L. showed no improvement in both HOMA-β and HOMA-IR. (4) Conclusions: *Ficus pumila* L. extract, ingested by patients who developed diabetes after COVID-19 infection, stimulated insulin secretion capacity and improved insulin resistance.

## 1. Introduction

A large study based on approximately 200,000 people found that patients infected with COVID-19 by SARS-CoV-2 have an increased risk of developing diabetes within a year, unrelated to the severity of symptoms upon infection [1,2].

It has been reported that patients with no previous risk factors for diabetes are also more likely to develop diabetes after infection. Patients who develop diabetes after infection resemble those with type 2 diabetes mellitus, and have been reported to have both impaired insulin secretion and increased insulin resistance, which are two major causes of type 2 diabetes mellitus.

**Figure 1:**
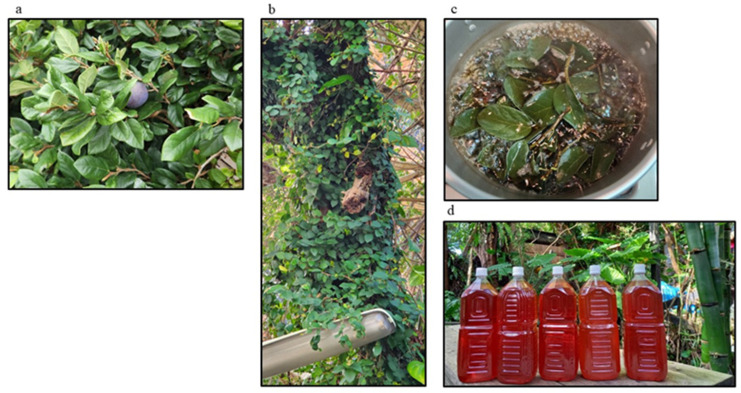
Close-up of the leaves, the extracts of Ficus pumila. (**a**) Leaves, stems, and fruits of *Ficus pumila* L. (**b**) *Ficus pumila* L. commonly known as the creeping fig or climbing fig around trees in nature is a species of flowering plant in the mulberry family. (**c**) Boiling washed leaves and stems of *Ficus pumila* L. in Motobu water. (**d**) *Ficus pumila* L. in the Motobu area of Okinawa Prefecture was carefully made into amber-colored Ficus pumila tea.

*Ficus pumila* L. is native to East Asia (China, Japan, and Vietnam) and belongs to the mulberry family in Figure 1a,b. *Ficus pumila* L. was named by the botanist Carl von Linne. In Japan, *Ficus pumila* L. is found only in Okinawa, where an internationally recognized area has outstanding longevity. The extracts of *Ficus pumila* L. have been traditionally consumed in Okinawa for more than 500 years to promote their health [3] in Figure 1c,d. In Okinawa, people drank tea made from the extraction of *Ficus pumila* L. plants for their health for more than 500 years. Now, this kind of tea is extremely rare.

In our study, *Ficus pumila* L. showed therapeutic efficacy in hypertension, hyperlipidemia, and hyperuricemia [3]. The reason for the use of *Ficus pumila* L. extract in the treatment of patients who developed diabetes after COVID-19 infection was that it was inferred that *Ficus pumila* L. has antiviral and antidiabetic effects [4,5]. We have not conducted enough research on normal people or people with diabetes who do not have COVID-19 and who are taking *Ficus pumila* L. extract. This is because if patients are taking antiglycemic medication or using insulin, there is a risk of hypoglycemia symptoms if patients ingest Ficus pumila L. extract. In this study, the history of oral use and insulin use are strictly excluded.

**Figure 2:**
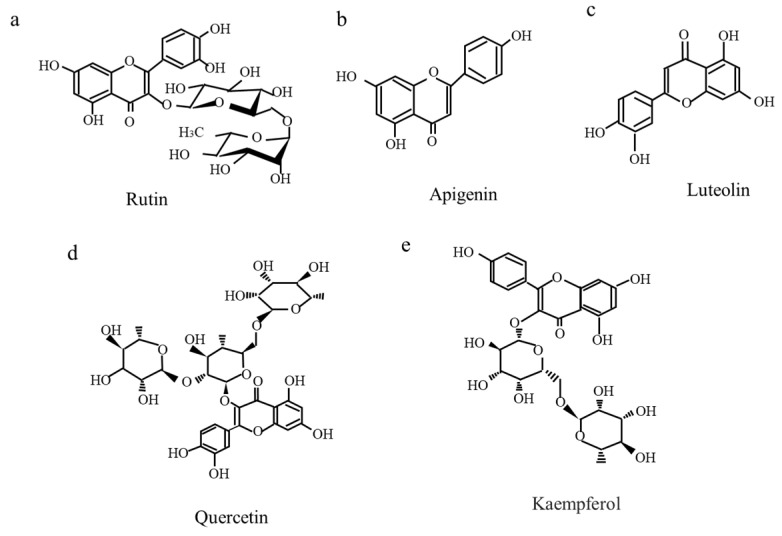
Bioactive polyphenol compounds. (**a**) Rutin, (**b**) Apigenin, (**c**) Luteolin, (**d**) Quercetin, and (**e**) Kaempferol have been extracted from *Ficus pumila* L. leaves and stems.

The bioactive compounds of *Ficus pumila* L. and the latest knowledge on treatments were reported in detail. Polyphenols (Rutin, Apigenin, Luteolin, Quercetin, Kaempferol), have so far been extracted from *Ficus pumila* L. leaves, stems, and fruits, and these bioactive compounds exhibit multiple therapeutic activities [6] in Figure 2a–e. However, it is now consumed only in certain areas of the main island, and its existence is unknown even to the islanders.

In the present study, we examined whether extracts from *Ficus pumila* L. are effective in improving the hyperglycemia of diabetic patients after COVID-19 infection.

## 2. Materials and Methods

### 2.1. Preparation of the Extract

The source of the *Ficus pumila* L. plant was the Motobu region of Okinawa Prefecture. *Ficus pumila* L. grows in the Motobu region of Okinawa Prefecture. Okinawa is a subtropical region, and plants grow well. Ficus pumila is no exception, and we can harvest *Ficus pumila* L. all year round. Collect fresh, undried branches and leaves that are wrapped around trees in nature in the Motobu region. Boil 200 g of the leaves and branches together in 2 L of water to remove the branches and leaves to extract the ingredients. The heating temperature is 100 degrees and the heating time is 10 min. The water is used from the soil deposited by old corals in the Motobu region of Okinawa. This method is a traditional method that has been passed down orally for 600 years. Patients living in the Motobu region of Okinawa Prefecture took 300 mL of extract per day. The leaves are finely ground and extracted with a 50% aqueous solution of ethanol. The solvent is removed, fractionated by column chromatography, and then 1H NMR measurement is performed. Rutin, Apigenin, Luteolin, Quercetin, and Kaempferol were detected. Especially two hundred grams of *Ficus pumila* L. branch and leaf contain approximately 2.44 mg and 1.41 mg of Rutin and Apigenin, respectively. The polyphenol content in the extract given to the individuals was inspected by the Okinawa Prefectural Environmental Science Center, which is a registered inspection agency of the Minister of Health, Labour and Welfare of Japan under the Food Sanitation Law. The polyphenol content given to the individual is 0.15 g per day. The total amount of polyphenols contained in *Ficus pumila* L. was measured by the Folin–Ciocalteu method.

### 2.2. Data on the Patients

One hundred and twenty-eight rehabilitation patients developed diabetes within 1 year after COVID-19 infection (June–October 2022). In total, 128 were mild to moderate COVID-19 patients. There was no significant difference regarding the severity of COVID-19 between the patients. In total, 128 were given the antiviral drug molnupiravir. 128 had residual diabetes six months later. A total of 128 patients of the participants had not taken *Ficus pumila* L. extract before they were enrolled in the study. In total, 128 patients had not taken insulin treatment or oral hypoglycemic medication. Of the 128 patients who were still diabetic after 6 months, 59 (31 women and 28 men, mean age: 81 years) were from the Motobu region of Okinawa Prefecture and had taken *Ficus pumila* L. extract or at least 3 months after developing diabetes. Of the 128 subjects, 69 (35 women and 34 men, mean age: 80 years) were not from the Motobu region and were still diabetic after 6 months; in total, 69 had not taken *Ficus pumila* L. extract. Blood samples were collected at 07:30 a.m. after overnight fasting to measure fasting plasma glucose and fasting insulin levels. In total, 128 patients were diagnosed with diabetes mellitus with an early morning fasting blood glucose level of 126 mg/dL or more, a 75 g oral glucose tolerance test (OGTT) 2 h blood glucose level of 200 mg/dL or more, an hourly blood glucose level of 200 mg/dL or more, and an HbA1c (NGSP) of 6.5% or more. In addition, the immune-reactive insulin (IRI) level was 2.5 or higher, and insulin resistance was diagnosed. Prior to infection, the 128 patients had blood glucose, HbA1c, and IRI levels all within the baseline range, and did not develop diabetes. There was also no use of insulin.

### 2.3. Method and Equipment

A Homeostatic model assessment of β-cell function (HOMA-β) and that of insulin resistance (HOMA-IR) were used. Values were calculated using a HOMA calculator, which was available on the Diabetes Trials Unit website (https://www.rdm.ox.ac.uk/about/our-clinical-facilities-and-units/DTU/software/homa, accessed on 8 January 2025).

The equations for HOMA-β and HOMAIR are as follows.

HOMA-β = Fasting insulin levels (μU/mL) × 360 ÷ (Fasting blood glucose (mg/dL) − 63)

HOMA-IR = Fasting blood glucose (mg/dL) × Fasting insulin levels (µU/mL)/405

HOMA-β is judged to be a decrease in insulin secretion capacity at 30% or less, and a marked decrease at 15% or less. HOMA-IR is judged to be insulin resistant at a time of 2.5 or higher. The HOMA-β and HOMA-IR were measured in 28 and 34 subjects.

Data are presented as means ± SD. *p* values were determined using Student’s *t*-test. A *p* value < 0.05 was considered significant. SAS software version 9.2 (SAS Institute Inc., Cary, NC, USA) was used for statistical analysis.

## 3. Results

**Figure 3:**
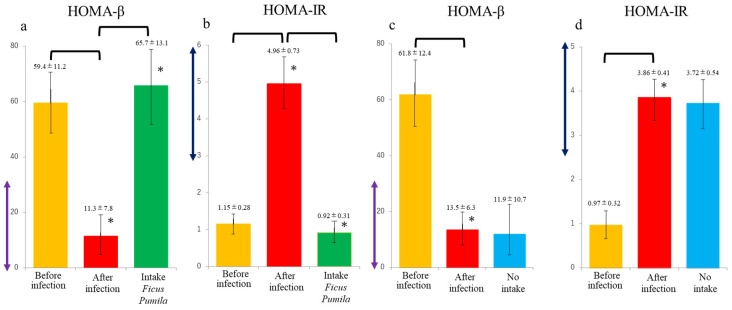
Results of HOMA-β and HOMA-IR evaluation. (**a**) Before COVID-19 infection, HOMA-β levels were within normal range. (orange box) HOMA-β levels decreased after infection (red box), but increased after consumption of *Ficus pumila* L. (green box). (**b**) Before COVID-19 infection, HOMA-IR levels were within normal range. (orange box). HOMA-IR evaluation after infection increased (red box), but decreased after ingestion of *Ficus pumila* L. (green box). (**c**) Before COVID-19 infection, HOMA-β levels were within normal range. (orange box) Post-infection HOMA-β levels decreased (red box) but continued to decrease. (blue box). (**d**) Before COVID-19 infection, HOMA-IR levels were within normal range. (orange box). Post-infection HOMA-IR levels increased (red box), but remained elevated. (blue box). Data are presented as average ± SD. * shows *p* < 0.05. HOMA-β levels: decreased insulin secretion capacity at 30% or less (violet two-way arrow). HOMA-IR levels: insulin resistance higher than 2.5 (purple two-way arrow).

HOMA-β value significantly decreased after COVID-19 infection in the 59 subjects, (before; 59.4 ± 11.2 and after: 11.3 ± 7.8 *p* < 0.05, Figure 3a). However, HOMA-β significantly improved to 65.7 ± 13.1 after 3 months of *Ficus pumila* L. extract intake (*p* < 0.05, Figure 3a). The HOMA-IR before and after COVID-19 infection were 1.15 ± 0.28 and 4.96 ± 0.73 (*p* < 0.05, Figure 3b), respectively. The HOMA-IR value of the *Ficus pumila* L. extract ingested group was also significantly improved to 0.92 ± 0.31 after 3 months (*p* < 0.05, Figure 3b). On the other hand, the pre- and post-infection HOMA-β values of the 69 control subjects without *Ficus pumila* L. extract ingestion were 61.8 ± 12.4 and 13.5 ± 6.3 (*p* < 0.05, Figure 3c), respectively. The value further decreased to 11.9 ± 10.7 (Figure 3c). The HOMA-IR before and after infection were 0.97 ± 0.32 and 3.86 ± 0.41 (*p* < 0.05, Figure 3d), respectively. The value further increased to 3.72 ± 0.54 (Figure 3d). Results of normal people or people with diabetes who do not have COVID-19 and who are taking *Ficus pumila* L. extract have not been obtained.

## 4. Discussion

A patient with diabetes mellitus after COVID-19 infection died after acute pancreatitis and diabetic necrosis. Even mild or moderate COVID-19 infection can become serious if diabetes is later developed. If non-diabetic patients develop diabetes due to COVID-19 infection, the mechanism should be elucidated and cured. Therefore, we focused on the efficacy of *Ficus pumila* L. extract, which has been ingested in Okinawa as a folk remedy since ancient times. In the present study, *Ficus pumila* L. extract, ingested by patients who developed diabetes after COVID-19 infection, stimulated insulin secretion capacity and improved insulin resistance.

### 4.1. Pathogenesis of the Development of Diabetes Mellitus in Coronavirus Infection

Inhibition of Neuropilin 1 (NRP1), which is expressed in pancreatic tissue, has been reported to improve glucose tolerance in type 2 diabetes as well as in glucose intolerance after SARS-CoV2 infection [7,8,9]. Proteins such as ACE2 co-receptors NRP2 may also be responsible for worsening the COVID-19 course although many mechanisms associated with Angiotensin I-converting enzyme type 2 (ACE2) can lead to increased SARS-CoV-2 virulence in diabetes mellitus [10]. The SARS-CoV-2 proteins interact with targets such as the ACE2 receptor. In addition, SARS-CoV-2 can stimulate pathological intracellular signaling pathways by triggering NRP1. ACE2 may also be involved in reduced insulin secretion after SARS-CoV-2 infection. Consistently, high ACE2 expression in the islets of type 2 diabetes patients is reported to increase their susceptibility to SARS-CoV-2 infection, leading to impaired glucose metabolism [11]. The S-glycoprotein (Spike) of the SARS-CoV-2 forms a complex with the human transmembrane protein ACE2 during infection. ACE2 is a host receptor for SARS-CoV-2. Inhibiting the interaction between the envelope Spike of SARS-CoV-2 and ACE2 is a potential antiviral therapeutic approach, but little is known about how dietary compounds interact with ACE2.

In this study, *Ficus pumila* L. consumption also improved insulin resistance as indicated by HOMA-IR. Insulin resistance is closely related to the expression of glucose transport (GLUT) receptors on the cell surface of peripheral organs such as skeletal muscle. In particular, there is a correlation between the expression of GLUT4 and the level of insulin resistance [12]. Reduced GLUT4 expression is one of the causes of developing insulin resistance in type 2 diabetes [13]. It has been reported that ACE2 expression, a SARS-CoV-2 receptor, decreases with infection in peripheral tissues and this may lead to a decrease in GLUT4 expression, which ultimately develops insulin resistance [14,15]. SARS-CoV-2 also binds to ACE2 expressed in skeletal muscle tissues and induces inflammatory effects, cytokinesis, and muscle catabolism, damaging the musculoskeletal system [16]. Injury of skeletal muscle fibers by SARS-CoV-2 infection has also been reported to decrease the expression of GLUT4 [17].

### 4.2. Antioxidant Targets in the Treatment of COVID-19 and Complications

The polyphenol compounds inhibited 50% of virus–receptor binding interactions for NRP1 and ACE2, respectively. Polyphenol compounds more significantly interacted with the NRP1 receptor. Especially Quercetin was a compound with the highest exhibited interfering potential for selected target receptors [18]. Polyphenol is a known NRP1,2 inhibitor, and since *Ficus pumila* L. also contains polyphenols, it is possible that the action of polyphenol in *Ficus pumila* L. may have normalized insulin secretory capacity in patients presented in this study. Polyphenols, which are also the ingredients of *Ficus pumila* L., inhibit recombinant human ACE2 activity, which may prevent the development of type 2 diabetes [19]. Polyphenols are the major phytoconstituents that display antidiabetic activity by interacting with key protein molecules related to the MAPK and PI3K-AKT signaling pathways, thereby aiding in the treatment of type 2 diabetes mellitus.

### 4.3. Arguments Related to the Consequences of Treating Diabetes Post-COVID-19 with Antioxidant Therapy According to the Literature

**Figure 4:**
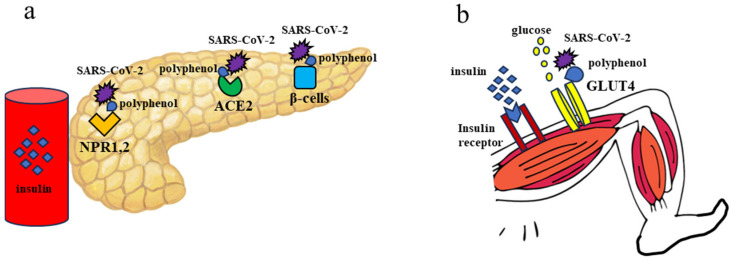
Pancreas and axial of femoral muscle. (**a**) Pancreas. Polyphenols protect the function of β-cells and inhibit Neuropilin 1, 2 (NPR1,2) and recombinant human ACE2 activity in pancreatic tissue infected with SARS-CoV-2. Insulin secretion deficiency improves, leading to increased secretion and increased amount of insulin in the blood. (**b**) Quadriceps femoris. Polyphenols stimulate GLUT4 expression, which is decreased by SARS-CoV-2 infection. Insulin and glucose bind to GLUT4 and are taken up by muscle cells. Insulin and glucose bind to GLUT4 and are taken up by muscle cells. Insulin resistance is improved, allowing insulin and glucose to be taken up into the muscles.

Rutin and Quercetin inhibited rhACE2 activity. In particular, Quercetin was the most potent rhACE2 inhibitor among the polyphenols tested [20]. Quercetin and Rutin had remarkable antiviral potential against different viral infections. Rutin is an antiviral drug with high binding affinity to proteins SARS-CoV-2, SARS-CoV, and SARS-CoV-2 Spike protein [21]. It has been reported that SARS-CoV-2 infection decreases insulin expression in β-cells and further induces apoptosis of β-cells, resulting in decreased insulin secretory capacity [22]. Polyphenols are also thought to have protective effects on pancreatic β-cells [23] in Figure 4a. Rutin and Quercetin are promising polyphenols that showed great activity against diabetes and diabetes complications in vitro and in vivo, induced glucose uptake via increasing GLUT-4 expression and/or translocation through insulin signaling pathway, AMPK pathway or acting as partial PPAR-γ agonists [24]. The Apigenin, Luteolin, and Kaempferol increased the expressions of PPAR-γ and GLUT4; they induced GLUT4 translocation [25]. Treatment with Kaempferol 3-O-rutinoside resulted in the upregulation of insulin-dependent p-IRS, AKT, and AMPK signaling molecules, and stimulation of the GLUT4 translocation, which ultimately enhanced the glucose uptake in insulin-resistant skeletal muscle myotubes [26]. GLUT4, AKT2, and AMPK were docked with Quercetin and Kaempferol. Hyperglycaemia and insulin sensitivity via activation of AKT2 and AMPK were ameliorated, and the expression of GLUT4, AKT2, and AMPK, whose levels were reduced under diabetic conditions, increased [27]. Polyphenols increased mRNA expressions of GLUT4 in the pancreas and demonstrated a good anti-diabetic profile by improving insulin sensitivity and GLUT-4 translocation [28] in Figure 4b.

The pathological mechanism of developing diabetes, after COVID-19 infection, is still unclear [29,30]. Due to the limited number of cases of diabetes caused by COVID-19, opinions are still divided [31]. However, the results of this study suggest that at least the point of action of *Ficus pumila* L. may be involved in the development of diabetes after COVID-19 infection, and the results may contain important insights not only into the mechanism of diabetes development after COVID-19 infection but also for the treatment.

## 5. Conclusions

*Ficus pumila* L. extract can improve insulin secretory capacity and insulin resistance in post-COVID-19 infection-induced diabetes.

## Data Availability

The original contributions presented in this study are included in the article. Further inquiries can be directed to the corresponding author.
